# Chromosome-Level Genome Assembly of the Burbot (*Lota lota*) Using Nanopore and Hi-C Technologies

**DOI:** 10.3389/fgene.2021.747552

**Published:** 2021-11-23

**Authors:** Dan Song, Yuting Qian, Minghui Meng, Xiaoli Dong, Wenqi Lv, Tangbin Huo

**Affiliations:** ^1^ Heilongjiang River Fisheries Research Institute, Chinese Academy of Fishery Sciences, Harbin, China; ^2^ Heilongjiang River Basin Fishery Ecological Environment Monitoring Center, Ministry of Agriculture and Rural Affairs, Harbin, China; ^3^ Institute of Hydrobiology, Chinese Academy of Sciences, Wuhan, China; ^4^ University of Chinese Academy of Sciences, Beijing, China; ^5^ Diggs (Wuhan) Biotechnology Co., Ltd., Wuhan, China

**Keywords:** *Lota lota*, chromosomal assembly, Nanopore, Hi-C, gene annotation, comparative genomics

## Introduction

Burbot (*Lota lota*) is the sole freshwater representative of the family Gadidae and represents the widest longitudinal distributed freshwater fish species worldwide. It is mainly distributed in rivers and lakes above 40° north latitude, including Eurasia and North America water systems (Cohen, et al., 1990). Burbot evolved from marine codfish to freshwater fishes about 10 million years ago and retained many characteristics of its marine ancestors, including cold-water preference, low-temperature spawning, high fecundity, and larval pelagic stage (Blabolil, et al., 2018). For instance, burbot spawns in winter or early spring with very low water temperatures (below 4°C), and the water temperature of above 5°C will be detrimental to the survival of burbot eggs (Żarski, et al., 2010). Therefore, burbot serves as a good model for adaptive evolution studies on both marine freshwater transition and cold-water preference.

Due to the stenothermal distribution, burbot is vulnerable to environmental changes and regarded as an excellent indicator for cold-water fish species (Stapanian et al., 2010a; Stapanian et al., 2010b). In recent decades, the burbot stocks and distribution have been severely decreasing due to overfishing, pollution, and habitat destruction (Li et al., 2020), which also resulted in lower genetic diversity, lower age, and miniaturization of burbot individuals. At present, many burbot populations have been threatened and endangered or even extirpated in some regions of North America and Eurasia (Stapanian et al., 2010a). Therefore, the appropriate management measures, such as the protection of habitat and spawning grounds, are essential for the population recovery of burbot. In the meantime, it is important to develop genomic resources to protect, restore, and effectively manage the natural resources of burbot.

Owing to its high levels of unsaturated fatty acids and various amino acids, burbot also has an important economic value, and it is famous for its delicious liver and testis of male fish. In recent years, various progresses of reproduction, larval domestication, and farming of burbots have been achieved (Yang, et al., 2021). A high-quality burbot genome is necessary for the genome-assisted breeding. Here, we constructed a high-quality chromosome-level genome assembly of *L. lota*, and the availability of reference genome will provide valuable resources for in-depth biological and evolutionary studies and genetic improvement of burbot.

## Data

In total, we generated about 52.99-Gb Nanopore long reads with an average read length of 20,705 bp, 66.13-Gb Illumina short reads, and 66.45-Gb Hi-C data for genome assembly (Supplementary Table S1). To estimate the main genome characteristics of *L. lota*, the k-mer-based method was applied, and 22, 444, 539,464 of 17-kmers were generated from the Illumina sequencing data. Finally, the *L. lota* genome size was estimated to be 565.64 Mb with 0.5% heterozygosity (Supplementary Table S2).

We used Nanopore long reads to construct the primary assembly, the size of which was 583.75 Mb with contig N50 of 15.29 Mb (Supplementary Table S3) after correction. To improve the accuracy of the assembly, a chromosome-level genome was constructed by clean Hi-C data. Based on the genome-wide Hi-C heatmap, the interacting signals around the diagonal was strong, and the 24 pseudochromosomes could be distinguished clearly, consistent with the karyotype results based on cytological observation (Kirtiklis, et al., 2016; Zhou, et al., 2019) (Supplementary Figure S1). The final genome assembly was 583.78 Mb, with a contig N50 of 9.08 Mb, and a scaffold N50 of 21.89 Mb (Table 1). In comparison with a recently released burbot genome assembly by Han et al. (2021), it was suggested that the genome assembled in this study has better quality for higher contig N50 and more accurate chromosome number (Supplementary Table 4). The contig N50 of the genome assembled by Han et al. (2021) was 2.01 Mb, and the assembled sequences were anchored to 22 pseudochromosomes, which might be attributed to a low resolution of Hi-C assembly or chromosome loss or fusion caused by local adaptation. A total length of 537.89 Mb of the genomic sequence was anchored, accounting for 92.1% of the entire assembly (Supplementary Table 5). Furthermore, the quality of the genome was evaluated by mapping short reads to genome and benchmarking universal single-copy ortholog (BUSCO v3) analysis (Simão, et al., 2015); 99.56% of short reads were mapped, which covered 99.88% of the assembled genome. The genome contained 3,452 (94.8%) complete BUSCOs, including 3,419 single-copy BUSCOs and 33 duplicated BUSCOs (Supplementary Table 6), indicating that the genome assembly had high completeness.

**TABLE 1:** Genome assembly statistics for the *Lota lota*.

Type	Scaffold	Contig
Total length (Mb)	583.78	
N50 length (bp)	21,891,956	9,082,484
N90 length (bp)	15,782,100	470,183
Maximum length (bp)	53,663,097	23,977,901
GC (%)	44.78	44.78

Note. GC, guanine–cytosine.

We identified a total of 332.58-Mb repeat sequences in the burbot genome, which accounted for 56.97% of the whole genome. The top three categories of repetitive elements were long terminal repeats (LTRs; 38.04%), simple sequence repeat (SSR; 8.36%), and DNA elements (8.00%) (Supplementary Table 7). A total of 21,672 protein-coding genes were annotated in the genome by three strategies as described in the materials and methods (Supplementary Table 8). Approximately 97.63% of the predicted genes were successfully annotated using five protein databases: National Center for Biotechnology Information (NCBI) Refseq (NR) (97.19%), EuKaryotic Orthologous Groups (KOG; 70.77%), Kyoto Encyclopedia of Genes and Genomes (KEGG; 67.96%), Gene Ontology (GO; 58.97%), and Swiss-Prot (94.44%) (Supplementary Table 9). Furthermore, noncoding RNAs were predicted across the burbot genome; and a total of 853 microRNAs (miRNAs), 7,588 transfer RNAs (tRNAs), 155 ribosomal RNAs (rRNAs), and 1,371 small nuclear RNAs (snRNAs) were detected (Supplementary Table 10).

**FIGURE 1:**
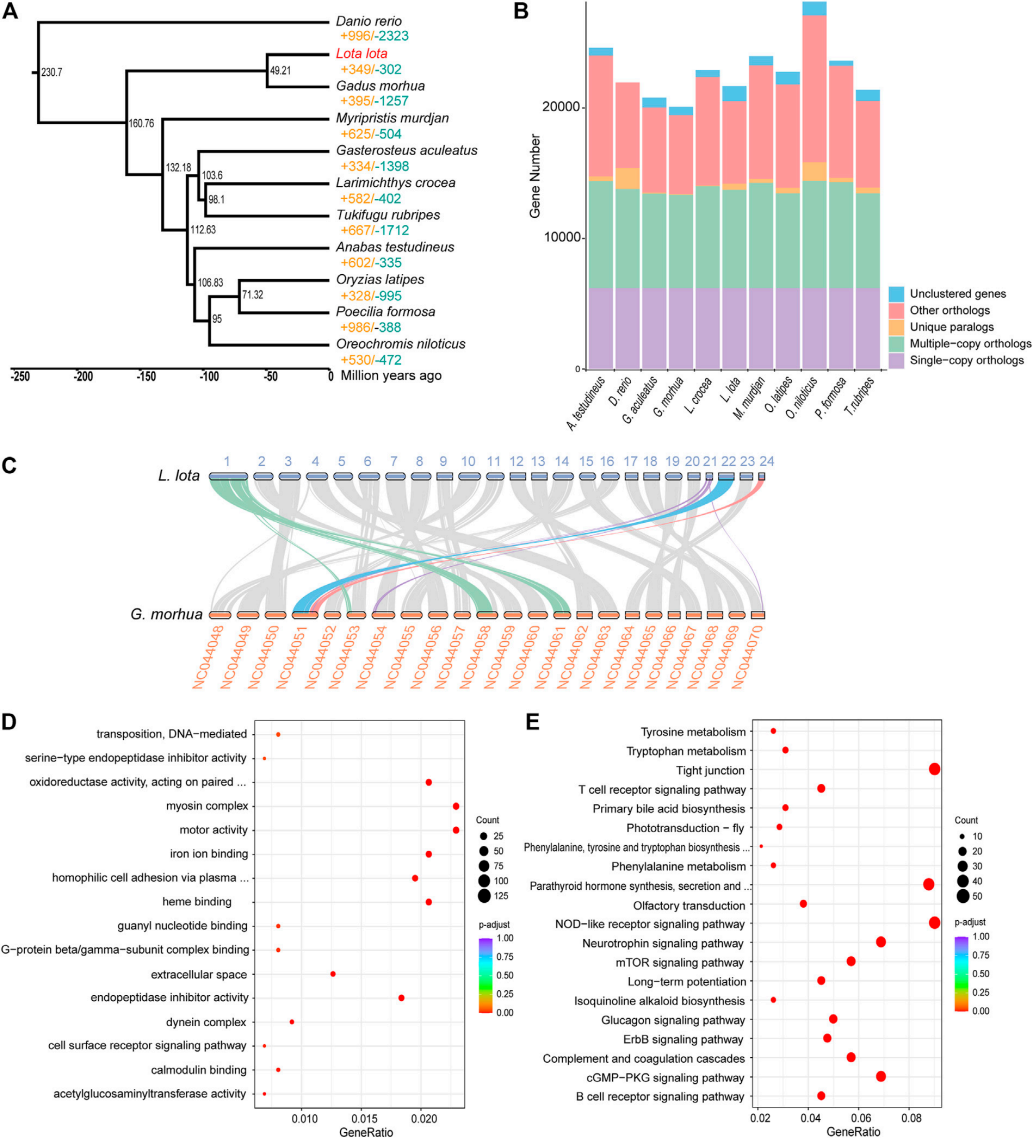
Phylogeny, orthologs, collinearity, and gene family evolution. **(A)** Phylogeny, dating, and gene family evolution. The value of significantly expanded (orange) and contracted (blue) gene families is designated on each branch. The estimated species divergence time (million years ago) is labelled at each branch site. **(B)** Statistics of orthologs and paralogs. “Others orthologs” indicates unclassified orthologs; “Unclustered genes,” orthologs that cannot be assigned into any orthogroups. **(C)** Collinearity analysis of *Lota lota* and *Gadus morhua* genomes. **(D)** Function enrichment of Gene Ontology (GO) for significantly expanded gene families. **(E)** Kyoto Encyclopedia of Genes and Genomes (KEGG) enrichment analysis for significantly expanded gene families. Only the top 20 categories are shown.

To investigate the phylogenetic relationships between *L. lota* and other teleosts, OrthoFinder v2.3.4 (Emms and Kelly 2015) was applied for ortholog group identification. A total of 7,524 gene families and 6,211 single-copy orthologs shared by *L. lota* and other fishes were identified. A total of 474 gene families were specific to *L. lota* (Figure 1B). We constructed a phylogenetic tree using 6,211 single-copy orthologs by Iq-TREE v2 (Minh, et al., 2020), which showed that Atlantic cod (*Gadus morhua*) was most closely related to *L. lota* with a divergence time around 49.21 million years ago (Figure 1A). Through synteny analysis between *G. morhua* and *L. lota*, we found that the chromosomes between *L. lota* and *G. morhua* were highly conserved (Figure 1C). We also detected that chromosome 1 of *L. lota* was compared with chromosomes NC044058 and NC044061 of *G. morhua*, and chromosomes 21 and 24 of *L. lota* were mapped to chromosome NC044051 of *G. morhua* (Figure 1C), which indicated chromosome fission and fusion events of the ancestral chromosomes.

The expansion and contraction of gene family are two of the most important factors for formation of special adaptive mechanisms during evolutionary process. We estimated the gene family evolution using CAFÉ v4.2.1 (Han et al., 2013); there were 349 and 302 gene families that experienced significant expansions and contractions for *L. lota*, respectively. The expanded genes were significantly enriched in 16 GO terms, mainly involved in endopeptidase inhibitor activity (GO:0004866, GO:0006313), homophilic cell adhesion *via* plasma membrane adhesion molecules (GO:0030286), and iron ion binding (GO:0005506) (Figure 1D, Supplementary Table 11). Meanwhile, functional enrichment analyses using KEGG database mapped expanded genes to 53 pathways, including immune-associated pathways, such as NOD-like receptor signaling pathway, complement and coagulation cascades, and T-cell receptor signaling pathway. In addition, the pathway involved the biological process regulation, mTOR signaling pathway, which was significantly enriched (Figure 1E, Supplementary Table 12).

## Materials and Methods

### Sample Collection and Sequencing

For genome sequencing and assembly, the muscle tissue of a male burbot was dissected, and genomic DNA was extracted using Qiagen GenomicTip100 (Qiagen, Hilden, Germany). To generate long reads used for genome assembly, we constructed a Nanopore 20-kb insert library with 1 µg of genomic DNA. The constructed library was sequenced by the Oxford Nanopore Technologies using PromethION sequencer. Then Illumina short reads were generated for base-level correction after assembly. A paired-end (PE) library with 500-bp insert size was constructed, and it was sequenced on Illumina HiSeq 4000 platform; finally, 150-bp PE reads were generated. To obtain a chromosome-scale genome, liver tissue from the same individual was used for Hi-C library preparation and sequencing. Firstly, the liver tissue was fixed in 1% formaldehyde solution to perform cross-linking. Nuclei were further obtained and digested with *Dpn*II, marked with biotin-14-dCTP, and then ligated by T4 DNA ligase. The DNA was extracted and sheared, the biotin-labelled DNA fragments were enriched, and finally, PE libraries with 500-bp insert size were constructed. The Hi-C libraries were sequenced on Illumina HiSeq 4000 platform with 150-bp PE mode.

With the same method used in short-read sequencing, for gene prediction, RNA-seq was performed with several tissues, including three liver, heart, brain, ovary, and testis tissues. We constructed cDNA libraries for each tissue sample and sequencing on Illumina platform.

### Genome Estimation and Genome Assembly

Before genome assembly, the k-mer analysis was conducted to estimate the genome size and heterozygosity of *L. lota* using Jellyfish v2.2.10 (Marçais and Kingsford 2011) and Genomescope v1.0 (Vurture, et al., 2017) based on Illumina short reads filtered by fastp v0.22.0 (Chen, et al., 2018). We corrected the Nanopore long reads by NextCorrect modules of NextDenovo v2.3.0 (https://github.com/Nextomics/NextDenovo) to assemble the primary genome using software wtdbg2 (Ruan and Li 2020). Three rounds of genome sequence polishing were performed to correct random sequencing errors using Pilon v1.23 (Walker, et al., 2014) with the cleaned short reads. For chromosome-level genome assembly, two ends of paired reads were independently aligned to the polished genome using Bowtie v1.2.2 (Langmead 2010), and only the read pairs that were uniquely mapped to the genome were selected. Last, the valid Hi-C read pairs were applied for clustering, ordering, and orienting to aid in anchoring the contig to the chromosomes using Lachesis (Burton, et al., 2013). Finally, to evaluate the quality of *L. lota* genome, the Illumina reads were mapped back to the genome using BWA-MEM v0.7.17 (Li and Durbin 2009) and the BUSCO analysis (Simão, et al., 2015) based on the actinopterygii_odb9 database performed.

### Genome Annotation

To annotate repeat sequences in the genome of burbot, we combined homology repeat prediction with *de novo* repeat prediction. TRF v4.09 (Benson 1999), RepeatMasker v4.06 (Tarailo‐Graovac and Chen, 2009), and RepeatProteinMask v4.06 were used for homology prediction by aligning the genome sequences against the RepBase library. For the second method, we employed RepeatModeller v1.08 and LTR-FINDER v1.06 (Xu and Wang 2007) based on the *de novo* repetitive element database.

Protein-coding genes of the genome were predicted based on *ab initio*, homology-based and transcriptome-based strategies. The *de novo* approach was conducted using Augustus v3.2.1 (Stanke, et al., 2008), Snap v2013-11-29 (Leskovec and Sosič 2016), GeneMark-EP+ (Bruna, et al., 2020), Geneid v1.4 (Alioto, et al., 2018), and GlimmerHMM v3.0.4 (Majoros, et al., 2004) based on the repeat-masked genome. For homology-based annotation, the protein sequences of (*Anabas testudineus*, *Danio rerio*, *Oryzias latipes*, *G. morhua*, *Poecilia formosa*, and *Myripristis murdjan*) were downloaded from Ensemble and aligned to the genome of burbot using TBlastN v2.8.1 (E-value ≤ 1e−05) (Altschul, et al., 1990). Genewise 2.4.1 (Birney, et al., 2004) was then used to identify accurate gene structures of alignment. For transcriptome-based prediction, the Illumina transcriptome sequence reads were aligned *via* Hisat2 v2.1.0 (Kim, et al., 2015); subsequently, StringTie v1.3.3b (Pertea, et al., 2015) was used to predict gene models. All gene models generated by the above methods were integrated with EVidenceModeler (EVM) v1.1.1 (Haas, et al., 2008). Functional annotation of the predicted genes was performed by searching public databases, including NR (Marchler-Bauer, et al., 2011), Swiss-Prot, KEGG (Kanehisa and Goto 2000), GO (Dimmer, et al., 2012), and KOG (Tatusov, et al., 2001) databases.

Noncoding RNAs, including miRNAs, snRNAs, tRNAs, and rRNAs were identified and annotated across the *L. lota* genome. The tRNAs were identified by program tRNAscan-SE v2.0.6 (Chan and Lowe 2019), and the highly conserved rRNAs were annotated using BlastN v2.8.1. Other ncRNAs were identified by searching against the Rfam database using Infernal v1.1.2 (Nawrocki and Eddy 2013).

### Phylogenomics and Gene Family Evolution

The protein sequences of 10 teleosts (*D. rerio*, *Gasterosteus aculeatus*, *Oreochromis niloticus*, *O. latipes*, *Takifugu rubripes*, *Xiphophorus maculatus*, *G. morhua*, *P. formosa*, *Larimichthys crocea*, and *M. murdjan*) were downloaded from Ensembl database (http://www.ensembl.org/index.html?redirect=no) (Supplementary Table S1), and the longest one was retained to represent each gene. Then the filtered sequences of the 10 species and *L. lota* were analyzed to construct different types of orthologs using OrthoFinder v2.3.4 (Emms and Kelly 2015). In order to study the evolutionary relationship, the single-copy protein sequences were used to construct the phylogenetic tree by Iq-TREE v2 (Minh, et al., 2020). We also estimated the divergence time through Bayesian relaxed molecular clock approach using MCMCTree in PAML v4.9j package (Yang 2007). Here, four soft-bound calibration times taken from www.timetree.org were applied: *D. rerio*–*G. aculeatus* (205–255 Mya), *G. morhua*–*G. aculeatus* (141–170 Mya), *M. murdjan*–*O. niloticus* (117–154 Mya), and *O. niloticus*–*O. latipes* (87–151 Mya). To visualize the consistency between the genomes of *L. lota* and its closely related species, *G. morhua*, the 24 *L. lota* chromosomes were aligned with *G. morhua* chromosomes by MCScanX (Wang et al., 2012).

We used the results from OrthoFinder and software CAFÉ v4.2.1 (Han, et al., 2013) to estimate expansion and contraction gene families among the 11 teleosts. The gene family with *p*-value <0.05 was thought to experience significant expansion or contraction. Then the significantly expanded families were functionally enriched by GO and KEGG enrichment analyses.

## Data Availability

The datasets presented in this study can be found in online repositories. The names of the repository/repositories and accession number(s) can be found in the article/Supplementary Material.
