# Supplementary material for: Chromosome-Level Genome Assembly of the Burbot (Lota lota) Using Nanopore and Hi-C Technologies
Source: Front Genet. 2021 Nov 23;12:747552. doi: 10.3389/fgene.2021.747552 (PMC8650494; doi:10.3389/fgene.2021.747552)
Supplement: Supplementary file 1 [file DataSheet1.docx]

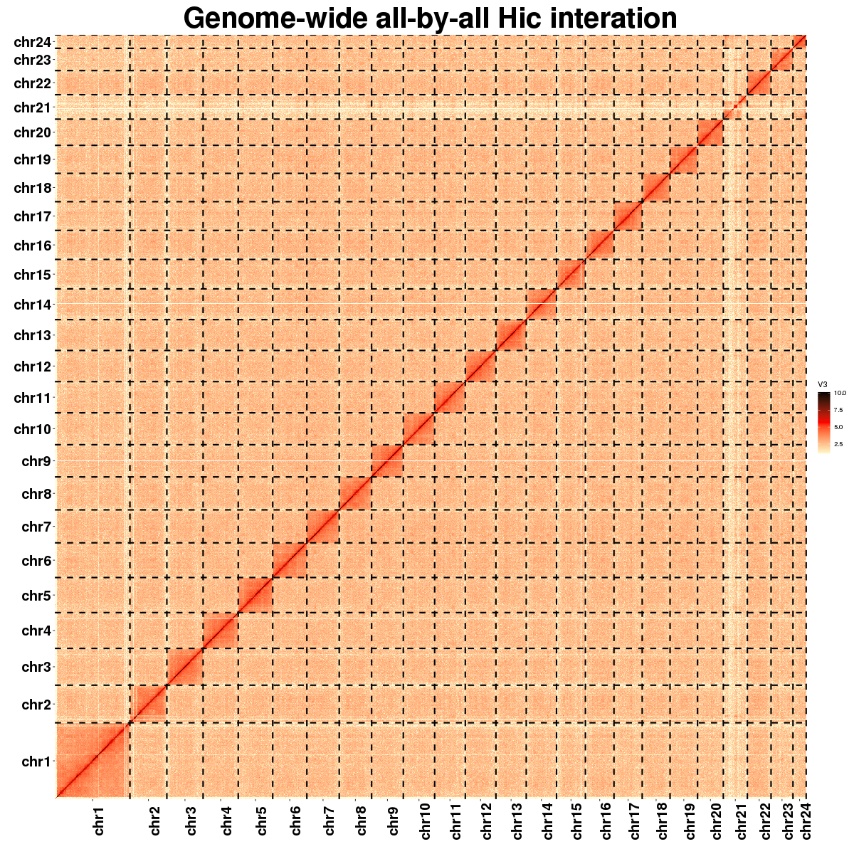


**Supplementary Figure 1.** Genome-wide Hi-C heatmap of *L. Lota*. The blocks represent the 24 pseudochromosomes. The color bar illuminates the contact density from yellow (low) to red (high).

**Supplementary Table 1.** Summary of obtained sequencing data generated from multiple sequencing technologies for *L. lota* genome assembly.

| **Sequencing libraries** | **Insert size** | **Clean data (Gb)** | **Read Length (bp)** |
| --- | --- | --- | --- |
| Nanopore | 20k | 52.99 | 20,705(Average) |
| Illumina | 500bp | 66.13 | 150 |
| Hi-C | 500bp | 66.45 | 150 |
| Total | - | 185.57 | - |

**Supplementary Table 2.** Estimation of genome size based on 17-mer statistics.

| Kmer | Depth | N-kmer | Genome size (Mb) | Heterozygous rate (%) |
| --- | --- | --- | --- | --- |
| 17 | 38.5725 | 22,444,539,464 | 565.6448 | 0.5 |

**Supplementary Table 3.** Statistics of *L. lota* genome assembly before Hi-C correction.

| Total length (Mb) | 583.75 |
| --- | --- |
| N50 length (bp) | 5,294,422 |
| N90length (bp) | 1,427,186 |
| Maximum length (bp) | 24,553,168 |
| GC (%) | 44.78 % |

**Supplementary Table 4.** The assembly statistic comparison between *L. lota* genome in this study and that published by Han et al. (Han et al. 2021).

|  | In this study | Han et al. 2021 |
| --- | --- | --- |
| Genome size (Mb) | 583.78 | 575.92 |
| ContigN50 (Mb) | 9.08 | 2.01 |
| ScaffoldN50 (Mb) | 21.89 | 22.10 |
| Number of pseudochromosomes | 24 | 22 |
| Loading Rate (%) | 92.1 | 88.66 |

**Supplementary Table 5.** Statistics of assembled 24 chromosomes of *L. lota* genome.

|  | Length(bp) | % of genome | Contig number |
| --- | --- | --- | --- |
| Chr1 | 53,659,397 | 0.092 | 38 |
| Chr2 | 26,352,463 | 0.045 | 32 |
| Chr3 | 25,933,549 | 0.044 | 12 |
| Chr4 | 25,119,859 | 0.043 | 3 |
| Chr5 | 24,683,859 | 0.042 | 4 |
| Chr6 | 24,482,901 | 0.042 | 2 |
| Chr7 | 23,169,000 | 0.039 | 4 |
| Chr8 | 23,167,000 | 0.039 | 2 |
| Chr9 | 22,668,780 | 0.039 | 4 |
| Chr10 | 22,356,855 | 0.038 | 1 |
| Chr11 | 21,890,856 | 0.037 | 12 |
| Chr12 | 21,884,890 | 0.037 | 4 |
| Chr13 | 21,781,859 | 0.037 | 4 |
| Chr14 | 21,760,890 | 0.037 | 5 |
| Chr15 | 20,618,370 | 0.035 | 11 |
| Chr16 | 20,566,578 | 0.035 | 20 |
| Chr17 | 20,167,324 | 0.034 | 10 |
| Chr18 | 19,927,254 | 0.034 | 6 |
| Chr19 | 19,677,890 | 0.034 | 2 |
| Chr20 | 18,542,791 | 0.0318 | 3 |
| Chr21 | 17,201,028 | 0.029 | 167 |
| Chr22 | 17,052,890 | 0.029 | 2 |
| Chr23 | 15,782,000 | 0.027 | 2 |
| Chr24 | 9,438,000 | 0.016 | 3 |
| Total | 537,886,283 | 0.921 | 353 |

**Supplementary Table 6.** The results of *L. lota* genomic quality assessment.

| BUSCO | Term | Number | Ratio (%) |
| --- | --- | --- | --- |
|  | Complete BUSCOS (C) | 3,452 | 94.8 |
|  | Single-copy BUSCOS (S) | 3,419 | 93.9 |
|  | Duplicated BUSCOS (D) | 33 | 0.9 |
|  | Fragmented BUSCOS (F) | 16 | 0.4 |
|  | Missing BUSCOS (M) | 172 | 4.8 |
| Short reads coverage | Percentage of mapped reads | - | 99.56 |
|  | Coverage (%) | - | 99.88 |

**Supplementary Table 7.** Summary statistics for the annotated repeat sequences.

| Repetitive sequences | Length (bp) | % of genome |
| --- | --- | --- |
| SINE | 2,058,015 | 0.35 |
| LINE | 11,416,625 | 1.96 |
| LTR | 222,033,348 | 38.04 |
| DNA | 46,675,539 | 8.00 |
| SSR | 48,786,939 | 8.36 |
| Total | 332,577,405 | 56.71 |

**Supplementary Table 8.** Summary statistics of predicted protein-coding genes.

| **Gene set** | **Number** | **Average gene length (bp)** | **Average CDS length (bp)** | **Average exons per genes** | **Average exon length (bp)** | **Average intros per gene** | **Average intro length (bp)** |
| --- | --- | --- | --- | --- | --- | --- | --- |
| **De novo** |  |  |  |  |  |  |  |
| Genemark | 94,175 | 3,090 | 784 | 11.16 | 70 | 10.16 | 226 |
| Augustus | 44,579 | 2,797 | 1,020 | 4.26 | 239 | 3.26 | 545 |
| Geneid | 18,614 | 12,461 | 1,222 | 7.62 | 160 | 6.62 | 1,698 |
| Genscan | 25,942 | 16,159 | 1,363 | 9.59 | 142 | 8.59 | 1,722 |
| Glimmhmm | 74,116 | 6,456 | 587 | 3.53 | 166 | 2.53 | 2,322 |
| SNAP | 32,276 | 22,035 | 1,448 | 9.24 | 156 | 8.24 | 2,499 |
| **Homolog** |  |  |  |  |  |  |  |
| *A. testudineus* | 27,788 | 8,121 | 1,354 | 6.81 | 199 | 5.81 | 1,165 |
| *D. rerio* | 25,905 | 8,032 | 1,355 | 6.71 | 201 | 5.71 | 1,168 |
| *G. morhua* | 27,677 | 7,373 | 1,204 | 6.6 | 182 | 5.6 | 1,101 |
| *M. murdjan* | 29,092 | 7,618 | 1,278 | 6.47 | 197 | 5.47 | 1,159 |
| *O. latipes* | 30,665 | 6,928 | 1,241 | 6.01 | 206 | 5.01 | 1,135 |
| *P. formasa* | 26,847 | 8,061 | 1,387 | 7.05 | 196 | 6.05 | 1,102 |
| **Evm** | 21,672 | 12,213 | 1.728 | 10.05 | 172 | 9.05 | 1,158 |

**Supplementary Table 9.** Statistics for the functional annotation of protein-coding genes.

| **Database** | **Gene Number** | **Percent (%)** |
| --- | --- | --- |
| KOG | 15,313 | 70.66 |
| KEGG | 14,729 | 67.96 |
| NR | 21,063 | 97.19 |
| SwissProt | 20,466 | 94.44 |
| GO | 12,779 | 58.97 |
| At least one database | 21,159 | 97.63 |
| Total | 21,672 | - |

**Supplementary Table 10.** Summary statistics of non-coding RNA annotation.

|  | **Type** | **Number** | **Average Length(bp)** | **Total Length(bp)** | **%Genome** |
| --- | --- | --- | --- | --- | --- |
| miRNA | - | 853 | 87 | 74,311 | 0.00922 |
| tRNA | - | 7,588 | 74 | 568,062 | 0.07048 |
| rRNA | 18S | 11 | 1,733 | 19,069 | 0.00237 |
|  | 28S | 11 | 4,308 | 47,391 | 0.00588 |
|  | 5.8S | 8 | 153 | 1,230 | 0.00015 |
|  | 5S | 125 | 115 | 14,404 | 0.00179 |
|  | Total | 155 | 529 | 82,094 | 0.01019 |
| snRNA | CD-box | 309 | 135 | 41,808 | 0.00519 |
|  | HACA-box | 137 | 142 | 19,577 | 0.00243 |
|  | splicing | 925 | 132 | 122,484 | 0.01520 |
|  | Total | 1,371 | 134 | 183,869 | 0.02281 |

**Supplementary Table 11.** Functional enrichment of GO for significantly expanded gene families.

| ID | Description | GO class | P-value | P-adjust | Count |
| --- | --- | --- | --- | --- | --- |
| GO:0004866 | endopeptidase inhibitor activity | MF | 5.57E-13 | 6.35E-11 | 16 |
| GO:0016705 | oxidoreductase activity, acting on paired donors, with incorporation or reduction of molecular oxygen | MF | 1.24E-07 | 4.06E-06 | 18 |
| GO:0003774 | motor activity | MF | 1.42E-07 | 4.06E-06 | 20 |
| GO:0016459 | myosin complex | CC | 1.42E-07 | 4.06E-06 | 20 |
| GO:0005506 | iron ion binding | MF | 3.83E-06 | 8.73E-05 | 18 |
| GO:0030286 | dynein complex | CC | 1.09E-05 | 0.00020 | 8 |
| GO:0005615 | extracellular space | CC | 1.31E-05 | 0.00021 | 11 |
| GO:0020037 | heme binding | MF | 2.27E-05 | 0.00032 | 18 |
| GO:0008375 | acetylglucosaminyltransferase activity | MF | 0.00011 | 0.00144 | 6 |
| GO:0007156 | homophilic cell adhesion via plasma membrane adhesion molecules | BP | 0.00032 | 0.00363 | 17 |
| GO:0005516 | calmodulin binding | MF | 0.00063 | 0.00658 | 7 |
| GO:0007166 | cell surface receptor signaling pathway | BP | 0.00126 | 0.01197 | 6 |
| GO:0019001 | guanyl nucleotide binding | MF | 0.00227 | 0.01840 | 7 |
| GO:0031683 | G-protein beta/gamma-subunit complex binding | MF | 0.00227 | 0.01849 | 7 |
| GO:0004867 | serine-type endopeptidase inhibitor activity | MF | 0.00466 | 0.03541 | 6 |
| GO:0006313 | transposition, DNA-mediated | BP | 0.00508 | 0.03620 | 7 |

**Supplementary Table 12.** Functional enrichment of KEGG for significantly expanded gene families.

| ID | Description | P-value | P-adjust | Count |
| --- | --- | --- | --- | --- |
| map04621 | NOD-like receptor signaling pathway | 8.96E-12 | 1.64E-09 | 38 |
| map04928 | Parathyroid hormone synthesis, secretion and action | 2.85E-11 | 2.61E-09 | 37 |
| map04610 | Complement and coagulation cascades | 2.02E-10 | 1.23E-08 | 24 |
| map00120 | Primary bile acid biosynthesis | 6.15E-10 | 2.81E-08 | 13 |
| map00950 | Isoquinoline alkaloid biosynthesis | 9.06E-09 | 3.32E-07 | 11 |
| map04722 | Neurotrophin signaling pathway | 1.38E-08 | 3.64E-07 | 29 |
| map00400 | Phenylalanine, tyrosine and tryptophan biosynthesis | 1.57E-08 | 3.64E-07 | 9 |
| map00360 | Phenylalanine metabolism | 1.59E-08 | 3.64E-07 | 11 |
| map04530 | Tight junction | 2.00E-07 | 4.07E-06 | 38 |
| map04662 | B cell receptor signaling pathway | 2.24E-06 | 4.10E-05 | 19 |
| map04720 | Long-term potentiation | 3.52E-06 | 5.45E-05 | 19 |
| map04745 | Phototransduction - fly | 3.57E-06 | 5.45E-05 | 12 |
| map00380 | Tryptophan metabolism | 3.90E-06 | 5.49E-05 | 13 |
| map04012 | ErbB signaling pathway | 4.86E-06 | 6.35E-05 | 20 |
| map00350 | Tyrosine metabolism | 2.26E-05 | 0.000276 | 11 |
| map04922 | Glucagon signaling pathway | 3.55E-05 | 0.000405 | 21 |
| map04740 | Olfactory transduction | 6.78E-05 | 0.00073 | 16 |
| map04660 | T cell receptor signaling pathway | 8.97E-05 | 0.000912 | 19 |
| map04150 | mTOR signaling pathway | 0.000175 | 0.001688 | 24 |
| map04022 | cGMP-PKG signaling pathway | 0.000262 | 0.002346 | 29 |
| map04011 | MAPK signaling pathway - yeast | 0.000269 | 0.002346 | 10 |
| map04725 | Cholinergic synapse | 0.000308 | 0.002469 | 22 |
| map00280 | Valine, leucine and isoleucine degradation | 0.00031 | 0.002469 | 11 |
| map04971 | Gastric acid secretion | 0.000343 | 0.002616 | 16 |
| map04611 | Platelet activation | 0.000421 | 0.003079 | 22 |
| map04066 | HIF-1 signaling pathway | 0.000571 | 0.004022 | 18 |
| map04114 | Oocyte meiosis | 0.000679 | 0.004303 | 18 |
| map04916 | Melanogenesis | 0.000679 | 0.004303 | 18 |
| map04310 | Wnt signaling pathway | 0.000682 | 0.004303 | 23 |
| map00270 | Cysteine and methionine metabolism | 0.000959 | 0.005847 | 11 |
| map04670 | Leukocyte transendothelial migration | 0.001003 | 0.005922 | 22 |
| map04361 | Axon regeneration | 0.001205 | 0.006892 | 17 |
| map04912 | GnRH signaling pathway | 0.001664 | 0.009229 | 16 |
| map04514 | Cell adhesion molecules (CAMs) | 0.00181 | 0.009614 | 22 |
| map04713 | Circadian entrainment | 0.001839 | 0.009614 | 19 |
| map04921 | Oxytocin signaling pathway | 0.002135 | 0.010853 | 24 |
| map04071 | Sphingolipid signaling pathway | 0.002459 | 0.012162 | 20 |
| map04261 | Adrenergic signaling in cardiomyocytes | 0.002828 | 0.013618 | 24 |
| map00250 | Alanine, aspartate and glutamate metabolism | 0.0036 | 0.016894 | 9 |
| map04020 | Calcium signaling pathway | 0.00464 | 0.021229 | 27 |
| map04015 | Rap1 signaling pathway | 0.005245 | 0.023351 | 29 |
| map04630 | Jak-STAT signaling pathway | 0.005487 | 0.023351 | 16 |
| map04925 | Aldosterone synthesis and secretion | 0.005487 | 0.023351 | 16 |
| map04640 | Hematopoietic cell lineage | 0.005684 | 0.02364 | 10 |
| map04370 | VEGF signaling pathway | 0.007159 | 0.029113 | 11 |
| map04728 | Dopaminergic synapse | 0.010663 | 0.04242 | 19 |
| map04270 | Vascular smooth muscle contraction | 0.011632 | 0.045291 | 18 |
| map04062 | Chemokine signaling pathway | 0.012505 | 0.047567 | 20 |
| map01040 | Biosynthesis of unsaturated fatty acids | 0.012736 | 0.047567 | 6 |
| map03460 | Fanconi anemia pathway | 0.01334 | 0.04808 | 8 |
| map03320 | PPAR signaling pathway | 0.013662 | 0.04808 | 11 |
| map04115 | p53 signaling pathway | 0.013662 | 0.04808 | 11 |

Reference:

Han Z, Liu M, Liu Q, Zhai H, Xiao S, Gao T. 2021. Chromosome‐level genome assembly of burbot (*Lota lota*) provides insights into the evolutionary adaptations in freshwater. Molecular Ecology Resources.
